# Multiple-Criteria Decision-Making and Sensitivity Analysis for Selection of Materials for Knee Implant Femoral Component

**DOI:** 10.3390/ma14082084

**Published:** 2021-04-20

**Authors:** Raman Kumar, Rohit Dubey, Sehijpal Singh, Sunpreet Singh, Chander Prakash, Yadaiah Nirsanametla, Grzegorz Królczyk, Roman Chudy

**Affiliations:** 1 Department of Mechanical Engineering, Guru Nanak Dev Engineering College, Ludhiana 141006, Punjab, India; sehgal91@yahoo.co.in (R.K.); sehijpalsingh@yahoo.in (S.S.); 2 Department of Mechanical Engineering, National University of Singapore, Singapore 119077, Singapore; snprt.singh@gmail.com; 3 School of Mechanical Engineering, Lovely Professional University, Phagwara 144411, Punjab, India; 4 Department of Mechanical Engineering, North Eastern Regional Institute of Science and Technology, Nirjuli 791109, Arunachal Pradesh, India; yaadudme@gmail.com; 5 Department of Mechanical Engineering, Opole University of Technology, 45-758 Opole, Poland; r.chudy@po.edu.pl

**Keywords:** knee arthroplasty, femoral implant materials, multi-criteria decision-making, degree of membership, sensitivity analysis

## Abstract

Total knee replacement (TKR) is a remarkable achievement in biomedical science that enhances human life. However, human beings still suffer from knee-joint-related problems such as aseptic loosening caused by excessive wear between articular surfaces, stress-shielding of the bone by prosthesis, and soft tissue development in the interface of bone and implant due to inappropriate selection of TKR material. The choice of most suitable materials for the femoral component of TKR is a critical decision; therefore, in this research paper, a hybrid multiple-criteria decision-making (MCDM) tactic is applied using the degree of membership (DoM) technique with a varied system, using the weighted sum method (WSM), the weighted product method (WPM), the weighted aggregated sum product assessment method (WASPAS), an evaluation based on distance from average solution (EDAS), and a technique for order of preference by similarity to ideal solution (TOPSIS). The weights of importance are assigned to different criteria by the equal weights method (EWM). Furthermore, sensitivity analysis is conducted to check the solidity of the projected tactic. The weights of importance are varied using the entropy weights technique (EWT) and the standard deviation method (SDM). The projected hybrid MCDM methodology is simple, reliable and valuable for a conflicting decision-making environment.

## 1. Introduction

With the development of technology, day by day, advanced and functional materials are being developed by researchers [1]. The biomaterials are used to create artificial organs for orthopaedic applications such as knee replacements, hip replacements, and orthopaedic accessories [2]. Total knee replacement (TKR) is a remarkable achievement in biomedical science to improve the length of human life [3,4,5]. The knee joint consists of four types of cruciate ligaments [6,7,8]. These cruciate ligaments act as a four-bar linkage mechanism in a parasagittal plane, and their functionality information is discussed briefly [9,10]. The knee mainly consists of three bone structures, namely, the femur, patella, tibia, and fibula [11,12,13,14]. TKRs mainly have three components: the femoral component, the tibial component, and the patellar component, i.e., the knee cap [8,15,16]. Biomaterials chosen must have some basic requirements for a femoral component of a TKR prosthesis, and these requirements may vary from one to another. The required properties for a femoral component of TKR are discussed here [1,5].

The success of TKR implantation depends upon the selection of optimal biomaterial. The commonly used biomaterials are cobalt-chromium (Co-Cr), SS 316L, NiTi alloy, and titanium (Ti) and its alloys [17,18,19,20]. All these materials possess different properties, which are discussed below.

Adequate strength: In a prosthetic knee joint, material strength plays a vital role in avoiding joint fracture. Under a loading condition, a malfunction of bone-implant articulation leads to the development of soft fibrous tissue that further results in more significant relative motion. After a while, the TKR components may have to be replaced by an artificial organ in revision surgery to eliminate pain and other inconvenience [1,5,17]. The weight and density of biological material for a knee implant need to be equivalent to that of bone. So, most of the time, specific strength can be utilized as the main parameter [1,5,18].

Elastic modulus: Stress shielding is the major problem occurred in the joint replacements, which depends upon the biomaterial elastic modulus. Due to this reason, the bone may get weak and degenerates the articulation of the knee implant and bone, which further results in the loosening and failure of the Knee implant [1,17]. The biomaterials must possess the low elastic modulus near to bone (15–30 GPa) to overcome the stress-shielding [1,5,18,19,20].

Ductility: Mechanical property can be utilized to express the extent to which material deformation is plastic in nature and devoid of any fracture known as ductility. It is essential to evade any brittle failure [1].

Corrosion resistance: In metallic biomaterials, corrosion is an inevitable concern because of corrosive body fluid. The leading cause of revision surgery in the case of TKR is corrosion, and it also leads to the reduction of implant life. The implants generally emit unwanted metallic ions, which are not biocompatible to the human body. These unwanted metal ions may dissolve in the human body’s transporting medium, which helps them either cumulate in tissues situated near the implant or move to the human body’s other organs. This may lead to a severe ailment, such as cancer, and may reduce human life [21]. Corrosion resistance must be considered while selecting the material for TKR.

Wear resistance: The leading cause of implant loosening is lower wear resistance or higher friction coefficients [1,22]. Moreover, the biological activeness of wear debris generates an unadorned inflammatory retort. All of this may cause damage to the healthy bone reinforcing the actual implant. Moreover, the friction creates corrosion, which is one of the substantial issues talked about earlier.

Biocompatibility: It is the function properties and characteristics of a substance being compatible with living tissue in specific situation is referred to as biocompatibility. When exposed to the body or bodily fluids, biocompatible materials do not create a toxic or immunological response. Cytotoxicity (cell-culture), sensitization assays, irritation tests, subchronic toxicity, genotoxicity, implantation tests, and hemocompatibility test has been most widely used to assess the biocompatibility of biomaterial through the use of cell lines in-vitro [1,23,24]. Cell culture assays determine the quantitative and qualitative-MTT assay cytotoxicity of biomaterials. Sensitization test determine the effect of chemical elements contained in biomaterials allergic or hypersensitivity reactions. The irritation tests measure the risk of local discomfort as a result of chemicals derived from a biomaterial. The Acute Systemic Toxicity test looks for leachable that cause systemic (rather than local) toxicity. The Subchronic toxicity tests are used to assess the potential for long-term or multiple exposures to biomaterials to cause harmful effects. Genotoxicity tests detect compounds that can cause irreversible and heritable genetic changes directly or indirectly through a range of mechanisms, using a set of in vitro and in vivo tests. Implantation study is used to determine the biocompatibility of biomaterials that directly contact host location.

Osseointegration: It is a fundamental requirement in orthopaedic, which is related to bone healing. Osseointegration refers to the structural and functional bonding between the living bone and the load-bearing implant’s surface without intervening soft tissue [17,25]. The biocompatibility of the implant material, the surface topography of the implant, the surgical procedure used, and the loading of the implants are all factors that affect the osseointegration process. In the over, the stability of implant is determined in terms of osseointegration, which can be measured in two stages. Mechanical contact with cortical bone provides primary stability. Biological stability is provided by secondary stability, which is accomplished by bone regeneration and remodelling. There are many approaches for determining implant stability. Invasive/destructive methods and non-invasive/non-destructive methods may be distinguished. A number of tests has been reported to test the osteointegration around the implantation [26,27]. Apart from this finite element analysis is also used to determine the osteointegration, which is measured in terms of micro-motion between implant and bone, and bone density regeneration around implants surface.

Cost: This is an essential factor in selecting the appropriate material for knee replacement because the cost of the material depends on its availability, machining, and transportation. The consideration of cost along with material properties is essential because of affordability factors among customers. 

Despite the high range of biomaterials, human beings are still suffering from knee-joint-related problems due to the inappropriate selection of TKR material. Initially, stainless steel has been used as a potential biomaterial for the orthopaedic applications, but low corrosion resistance restricted its use for implant application. After that, researchers acknowledge the use of cobalt-based biomaterials for orthopaedic application especially for knee joint replacements [28]. The major drawback of Co-based alloy has high elastic modulus (220 GPa) as compared to bone (15–30 GPa), which leads to stress-shielding and results in implantation failed. After that researcher identified the commercially pure titanium-based (CP-Ti) biomaterial for orthopaedic application. But CP-Ti possessed elastic modulus (165 GPa) more than bone and high content impurity makes the alloy unsuitable for knee implantation. Most widely used Ti-based alloy was Ti-6L-4V, which is also have several drawbacks such as low hardness, poor wear resistance, and Al/V ions releases in the host body that creates allergic reaction [29]. A number of high strength and low elastic Ti-based alloys has been developed by researchers for knee and orthopaedic application, which have own advantages and disadvantages. To overcome this problem, the optimum material selection for a knee prosthesis becomes vital [30]. Therefore, because of these properties possessed by different biomaterials, eleven different materials were chosen according to their availability.

Various MCDM methods are utilized to select different materials for different applications. The VIKOR and TOPSIS techniques were applied to pick gate dielectric material [31]. Different techniques of MCDM were used to select the best penstock material for hydropower plants, where four alternatives, namely, polyvinyl chloride (PVC), high-density polyethylene (HDPE), glass-reinforced polymer (GRP) and mild steel (MS), were considered, along with five attributes (yield strength, life, thickness, cost of material and maintenance cost), in the study. The result showed that TOPSIS and modified TOPSIS methods are best suited for penstock material selection, and mild steel is the best material compared to other materials [32]. The TOPSIS method, combined with the entropy technique, was used to pick freight transportation, as research showed primary transport logistics attributes and the order preference by similarity ideal solution (TOPSIS) algorithm were the preferred MCMO model for comparatively ranking alternative freights. The entropy weight technique minimizes the subjectivity in the selection of the weight of the attribute. This study combined the entropy weight technique with TOPSIS to improve freight selection decisions [33]. A conveyor selection problem was solved with six conflicting criteria and eight alternatives using WASPAS, MOORA, CODAS, and EDAS methods, and the results were validated with the Spearman coefficient. The study showed that CODAS, EDAS and WASPAS were in amicable agreement [34]. The factor relationship technique was introduced to assign weights, and the hard-magnetic material selection problem was solved by the WASPAS method [35]. The EDAS method is a newly established MCDM technique by Keshavarz Ghorabaee [36]; it is steady in diverse weights and reliable with other procedures such as VIKOR, TOPSIS, SAW, and COPRAS. EDAS has been applied in various construction and industrial applications. An extended EDAS method was utilized for supplier selection [37]. EDAS and TOPSIS were used to select biomass material while assigning weights of significance with fuzzy analytical hierarchy process (AHP) with seven biomass alternatives and seven conflicting criteria. Out of these, sugarcane bagasse ranked at the top of all other options [38]. The TOPSIS method was applied to choose a vacuum cleaner, considering twenty-six different models of eight brands. Karcher WD 3.200 came out as the first choice, followed by Karcher WD 4.200 and Eureka Forbes Sensi. Additionally, the study proved the benefits of MCDM according to customer, retailer and wholesaler points of view [39]. The WASPAS method was used to select a portable hard disk drive from five different brands available on the Indian market, with twenty-four alternatives, which shows the robustness of MCDM methodologies in a wide range of other option weightage environments, from the equal weight method to the standard deviation method. The results showed that Western Digital was the best brand out of the other four, as the top three models were from this brand in both weightage criteria [40]. The EDAS method was applied to select an inverter technology air conditioner from 11 different brands, and cost, power input, number of convenience features, airflow, annual energy consumption, and ISEER were the conflicting attributes [41]. WSM and WPM techniques were utilized to choose a mobile phone [42]. The turning variables were optimized concurrently using VIKOR, AHP and multi-attribute decision-making techniques [43,44]. A review of the literature reveals that MCDM methods are frequently used for selection purposes. A femoral component of TKR was selected using the VIKOR technique, considering 10 alternate materials, and, reportedly, porous and dense NiTi shape memory alloys were ranked first and second, respectively [1]. The fuzzy analytical hierarchy process (FAHP), with the preference ranking organization method for enrichment evaluations (PROMETHEE), was applied to select a femoral component for TKR [4]. 

A number of methods exist, based on MCDM, to assign ranks to various alternatives. These methods find the ranks of the options based on different computational principles. Hence, it becomes tricky for the end-user to choose an MCDM method. There is a need to inspect the procedure that can cartel the diverse MCDM methods’ ranks. The paper’s main objective is to explore and develop an MCDM hybrid approach for selecting the best femoral component of TKR and sensitivity analysis while varying weights of significance with objective preference using entropy and standard deviation weight methods.

## 2. Hybrid Decision-Making Methodology with Objective Preferences and Degree of Membership (DoM)

**Figure 1:**
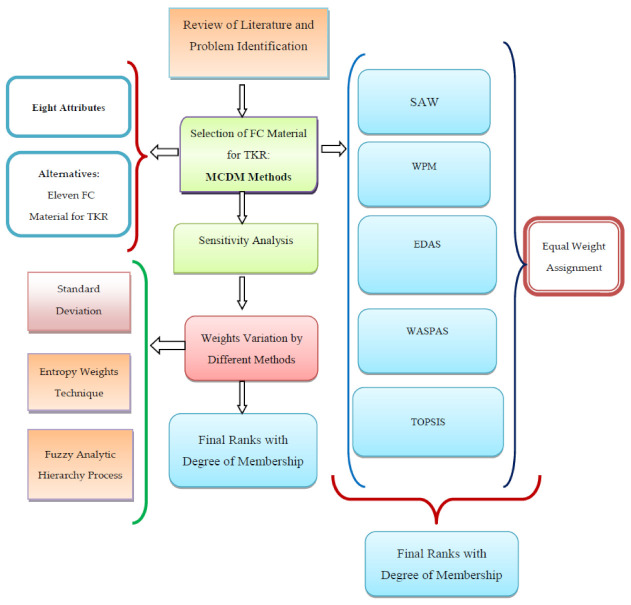
Proposed hybrid decision-making methodology.

The proposed MCDM, using a hybrid approach, is shown in Figure 1. First of all, the literature is reviewed after assessing a problem. This approach consists of five different MCDM methods, and these methods compute the final composite score based upon different principles. The ranks of alternatives are calculated with the equal weight method. The degree of membership (DoM) technique combines the ranks of FC of TKR alternatives. The working principle of the applied MCDM method is very clear from their names.

- The weighted sum method computes a preference score by taking an average of a normalized weighted matrix.
- The weighted product method is based on a geometric mean. The weighted aggregated sum product assessment method combines the results of WSM and WPM.
- The EDAS method computes a preference score using an evaluation based on distance from the average solution.
- The TOPSIS method is based upon a technique for order of preference by similarity to the ideal solution.
- The ranks obtained by the different MCDM methods are combined by DoM [45].
  Furthermore, the sensitivity analysis is executed by considering objective weights.
- Standard deviation method.
- Entropy weight technique.

The steps of multicriteria decision-making using the hybrid approach are as follows:

**Step 1:**

Identification of study objectives, alternatives, and attributes/criteria. The decision matrix ‘DM’ is considered, as per Equation (1). Every row of the decision matrix (DM) is assigned to each alternative (material) and each column to one attribute/criteria viz. cost, density, modulus of elasticity, tensile strength, elongation, corrosion resistance, wear resistance, and osseointegration. q_ij_ is an element of the decision matrix ‘DM’ [q_ij_; i = 1, 2, …, a number of alternatives (n), j = 1, 2, …, number of attribute/criteria (m)], which are inputs [46,47].

(1) $$DM=\left[\begin{array}{c} \begin{array}{ccc} q_{11} & q_{12} & \_\;\_\\ q_{21} & q_{22} & \_\;\_\\ \_\;\_ & \_\;\_ & \_\;\_ \end{array}\\ \begin{array}{ccc} q_{i1} & q_{i2} & \_\;\_\\ \_\;\_ & \_\;\_ & \_\;\_\\ q_{n1} & q_{n2} & \_\;\_ \end{array} \end{array}\begin{array}{c} \begin{array}{ccc} q_{1j} & \_\;\_ & q_{1m}\\ q_{2j} & \_\;\_ & q_{2m}\\ \_\;\_ & \_\;\_ & \_\;\_ \end{array}\\ \begin{array}{ccc} q_{\mathrm{ij}} & \_\;\_ & q_{\mathrm{im}}\\ \_\;\_ & \_\;\_ & \_\;\_\\ q_{\mathrm{nj}} & \_\;\_ & q_{\mathrm{nm}} \end{array} \end{array}\right].$$

**Figure 2:**
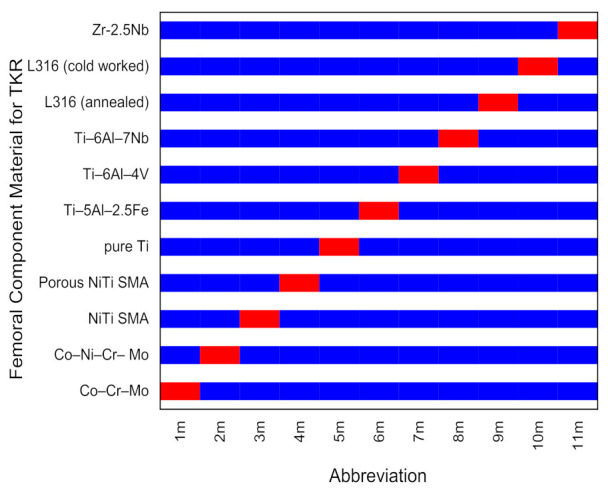
Applicant Femoral Component Material for TKR.

The detailed process involved in the calculation of ranks is shown in Figure 2. This involves literature review and problem identification at an early stage and the estimation of different ranks using Step 3 and Step 4, taking into consideration 8 attributes and 11 materials. The ranks are combined using Step 5. Finally, sensitivity analysis is done using three different weights according to Step 3, and a comparison is made using a graphical method.

**Step 2:**

The normalization of the decision matrix ‘DM’ is performed by different methods. The vector normalization technique is utilized by the ‘TOPSIS method’ and is shown in Equation (2). The linear-ratio-based normalization method is used by the ‘SAW method’, the ‘WPM method’, and the ‘WASPAS method’. It is shown in Equation (3) for beneficial attributes; nonbeneficial attributes are represented by Equation (4).

(2) $$M_{ij}=\frac{q_{ij}}{\sqrt{\sum_{i=1}^{n}q_{ij}{}^{2}}}$$

(3) $$M_{ij}=\frac{q_{ij}}{Max\;x_{ij}}\;\left(Beneficial\right).$$

(4) $$M_{ij}=\frac{Min\;q_{ij}}{q_{ij}}\;\left(Non-Beneficial\right)$$

**Step 3:**

There are various methods of assigning weights (w_j_) of significance to the attributes/criteria, (w_j_; j = 1, 2…… m, such that $\sum w_{j}$ = 1), such as equal, objective, and subjective preferences.

***Equal Weights Method (EWM)***

Equal weights are obtained by Equation (5).

(5) $$w_{j}=\frac{1}{m}$$

where *m* is the number of attributes.

***Standard Deviation Method (SDM)***

The SDM weights of the criteria are assessed by Equation (6) without taking into consideration the decision-maker’s subjective liking [48].

(6) $$w_{j}=\frac{\sigma_{j}}{\sum_{j=1}^{m}\sigma_{j}}$$

where $\sigma_{j}$ is the standard deviation of the dimensionless criteria.

***Entropy Weights Technique (EWT)***

The probability of the response ${(\mathrm{Pr}}_{\mathrm{ij}})$ happening, be computed by Equations (7) and (8), is utilized to attain the entropy (${\mathrm{En}}_{j})$ of the jth response [46,49].

(7) $${\mathrm{Pr}}_{\mathrm{ij}}=\frac{{\mathrm{NDM}}_{\mathrm{ij}}}{\sum_{i=1}^{n}{\mathrm{NDM}}_{\mathrm{ij}}}.$$

(8) $${\mathrm{En}}_{j}=-Y\overset{n}{\underset{i=1}{\sum }}{\mathrm{Pr}}_{\mathrm{ij}}\log_{e}{(\mathrm{Pr}}_{\mathrm{ij}}).$$

where $Y=\frac{1}{\log_{e}\;(n)}$ is a stable expression, n belongs to the number of experiments and the value of ${\mathrm{En}}_{j}$ lies between zero and one.

Equation (9) is utilized to compute the degrees of divergence (Div_j_), and Equation (10) obtains the entropy weight (E_w_) of the j^th^ response.

(9) $${\mathrm{Div}}_{j}=|{1\;-\;\mathrm{En}}_{j}|$$

(10) $$Ew_{j}=\frac{{\mathrm{Div}}_{j}}{\sum_{j=1}^{m}{\mathrm{Div}}_{j}}$$

**Step 4:**

Different types of methods have been used to find out the ranks of given alternatives. The techniques used in the present work are as follows:

***SAW (Simple Additive Weighted Method)***

The weighted normalized matrix (ŴŻ_ij_) is obtained by multiplying the columns of M_ij_ with their respective assigned weight, w_j_. Subsequently, ŴŻ_ij_ is attained by Equation (11).

(11) $$Ŵ{Ż}_{\mathrm{ij}\;}=\left[w_{j}{\;\times \;M}_{\mathrm{ij}}\right]$$

The criteria of optimality are applied based upon simple additive weighting (SAW), as shown in Equation (12).

(12) $$Q_{i}^{SAW}=\overset{m}{\underset{j=1}{\sum }}(w_{ij}\times w_{j})$$

***WPM (Weighted Product Method)***

The optimality criteria are applied based upon the weighted product method (WPM), as shown in Equation (13).

(13) $$Q_{i}^{WPM}=\overset{m}{\underset{j=1}{\prod }}{\left(M_{ij}\right)}^{w_{j}}$$

***WASPAS (Weighted Aggregated Sum Product Assessment)***

The dual comparative significance of the alternatives, i.e., performance index (*Q_i_*) based upon SAW and WPM techniques, is calculated, as shown in Equation (14) [50].

(14) $$Q_{i}^{WASPAS}=ƛQ_{i}^{SAW}+\left(1-ƛ\right)Q_{i}^{WPM}$$

and Equation (15) is used to locate the optimal assessment of ƛ for a specified decision-making problem.

(15) $$ƛ=\frac{\sigma^{2}Q_{i}^{WPM}}{\sigma^{2}Q_{i}^{WPM}+\sigma^{2}Q_{i}^{SAW}}$$

***EDAS (Evaluation Based on Distance from Average Solution)***

Determine the average solution (AV_ij_) according to all the criteria, as shown in Equation (16). The positive–negative-distance-based normalization is utilized in the EDAS method. The positive distance from average ($𝒫𝒟{𝒜}_{ij}$) is shown in Equation (17) for beneficial attributes and in Equation (18) for nonbeneficial attributes. The negative distance from average $𝒫𝒟{𝒜}_{ij})$ is shown in Equation (19) for beneficial attributes and in Equation (20) for nonbeneficial attributes [36].

(16) $$AV_{ij}=\frac{\sum_{i=1}^{n}q_{ij}}{n}$$

(17) $$𝒫𝒟{𝒜}_{ij}=\frac{\max\;\left(0,\;\left(q_{ij}-AV_{ij}\right)\right)}{AV_{ij}}\left[Beneficial\right]$$

(18) $$𝒫𝒟{𝒜}_{ij}=\frac{\max\;\left(0,\;\left(AV_{ij}-q_{ij}\right)\right)}{AV_{ij}}\left[Nonbeneficial\right]$$

(19) $$𝒩𝒟{𝒜}_{ij}=\frac{\mathrm{Max}\;\left(0,\;\left(AV_{ij}-q_{ij}\right)\right)}{AV_{ij}}\;\left[Beneficial\right]$$

(20) $$𝒩𝒟{𝒜}_{ij}=\frac{\max\;\left(0,\;\left(q_{ij}-AV_{ij}\right)\right)}{AV_{ij}}\;\left[Nonbeneficial\right]$$

The weighted sum of 𝒫𝒟𝒜 and 𝒩𝒟𝒜 is obtained from the average matrix from Equations (21) and (22).

(21) $$SP_{i}=\overset{m}{\underset{j=1}{\sum }}w_{j}\times 𝒫𝒟{𝒜}_{ij}.$$

(22) $$SN_{i}=\overset{m}{\underset{j=1}{\sum }}w_{j}\times 𝒩𝒟{𝒜}_{ij}.$$

The normalized values of $SP_{i}$ are obtained from Equation (23) and $SN_{i}$ from Equation (24) for all alternatives:

(23) $$NSP_{i}=\frac{SP_{i}}{Max_{i}\left(SP_{i}\right)}$$

(24) $$NSN_{i}=1-\frac{SN_{i}}{Max_{i}\left(SN_{i}\right)}$$

where $NSP_{i}$ and $NSN_{i}$ denote the normalized weighted sum of 𝒫𝒟𝒜 and 𝒩𝒟𝒜, respectively.

The appraisal score *AS_i_* for all alternatives is obtained by Equation (25):

(25) $$AS_{i}=\frac{1}{2}\;\left(NSP_{i}+NSN_{i}\right)$$

where 0 ≤ *AS_i_* ≤ 1.

***TOPSIS (Technique for Order Preference by Similarity to Ideal Solution)***

The weighted normalized matrix (ŴŻ_ij_) is obtained by multiplying the columns of M_ij_ with their particular allocated weight, w_j_. Subsequently, ŴŻ_ij_ is attained by Equation (26) [47,48].

(26) $$Ŵ{Ż}_{\mathrm{ij}}=\left[w_{j}{\;\times \;M}_{\mathrm{ij}}\right]$$

The ideal best (Ż+) and ideal worst (Ż−) solutions are computed by Equations (27) and (28), respectively. Here, Ż+ and Ż− solutions are the highest and least values amid all attribute values, respectively.

(27) $${Ż}_{j}^{+}=\{\mathrm{best}\;(Ŵ{{Ż}_{\mathrm{ij}})\}}_{i=1}^{n}\;{Ż}^{+}=\left\{{Ż}_{1}^{+}{,\;Ż}_{2}^{+}{,\ldots ,Ż}_{j}^{+}{,\ldots Ż}_{m}^{+}\right\}$$

(28) $${Ż}_{j^{\prime }}^{-}=\{\mathrm{worst}\;(Ŵ{Ż}_{{\mathrm{ij}}^{\prime }}{)\}}_{i=1}^{n}\;Ż=\left\{{Ż}_{1}^{-}{,\;Ż}_{2}^{-}{,\ldots ,\;Ż,\ldots Ż}_{m^{‘}}^{-}\right\}$$

where j and j′ are concerned with the beneficial (m) and nonbeneficial attributes (m′), respectively.

Prepare separation measures (Sep) with the assist of Euclidean distance (refer to Equations (29) and (30)).

(29) $${\mathrm{Sep}}_{i}^{+}=\{\overset{m}{\underset{j=1}{\sum }}{{(Ż}_{\mathrm{ij}}{\;-\;Ż}_{j}^{+})}^{2}{\}}^{0.5}$$

(30) $${\mathrm{Sep}}_{i}^{-}=\{\overset{m^{\prime }}{\underset{j^{\prime }=1}{\sum }}{{(Ż}_{\mathrm{ij}}{\;-\;Ż}_{j^{\prime }}^{-})}^{2}{\}}^{0.5}.$$

Compute the relative closeness or multiple composite score ‘MCS’ of all options, i.e., alternatives representing the equation’s ideal resolution refer to Equation (31).

(31) $$\mathrm{MCS}=\frac{{\mathrm{Sep}}_{i}^{-}}{{\mathrm{Sep}}_{i}^{+}{+\mathrm{Sep}}_{i}^{-}}$$

The relative closeness or ‘MCS’ achieved is then ordered into descending order for the ranking of alternatives.

**Step 5:**

This is the method to select optimal total knee replacement material using the final ranks of alternatives based on individual results from different MCDM methods [46]. 

Let $R_{𝓍𝓎}$ be the rank matrix of the ${𝓎}^{th}$ alternative using the ${𝓍}^{th}$ MCDM method (𝓍 = 1, 2, …, *k*, 𝓎 = 1, 2, ……, *t*), where *k* is the number of MCDM methods and *t* is the number of alternatives.

Step 5.1: Constitute the rank matrix R = (${𝓏}_{𝓍𝓎}$) *k* × *t*.

Step 5.2: Estimate the values of the rank variables; 𝓍 = 1, 2, …, *k*, 𝓎 = 1, 2, …, *t*, 𝓏 = 1, 2, ……, *t* from the rank matrix R = (${𝓻}_{𝓍𝓎}$) *k* × *t*, as shown in Equation (32).

(32) $$\delta_{𝓎𝓏}^{\left(𝓍\right)}=\left\{\begin{array}{c} 1;\;{𝓻}_{𝓍𝓎}=𝓏\\ 0;\;{𝓻}_{𝓍𝓎}\neq 𝓏 \end{array}\right.\;(𝓍=1,2,...,k,\;𝓎=1,2,...,t\;𝓏=1,2,\ldots ..,t$$

Step 5.3: Constitute rank frequency number matrix F = ($f_{𝓎𝓏}$) txt, where $f_{𝓎𝓏}$ is the rank frequency number so that the rank of the ${𝓎}^{th}$ alternative is ${𝓏}^{th}$ place by different MCDM methods, and $f_{𝓎𝓏}$ is calculated as per Equation (33).

(33) $$f_{𝓎𝓏}=\overset{k}{\underset{𝓍=1}{\sum }}\delta_{𝓎𝓏}^{\left(𝓍\right)}\;\left(,\;𝓎=1,2,\ldots ,t\;𝓏=1,2,\ldots \ldots ,t\right)$$

Step 5.4: Constitute membership degree matrix φ = ($\varphi_{𝓎𝓏}$) txt, where $\varphi_{𝓎𝓏}\;$ is the membership degree that the rank of the ${𝓎}^{th}$ alternative belongs to ${𝓎}^{th}$ place by different MCDM methods, and $\varphi_{𝓎𝓏}$ is as per Equation (34).

(34) $$\varphi_{𝓎𝓏}\;=f_{𝓎𝓏}/k\;(𝓎=1,2,\ldots ,t\;𝓏=1,2,\ldots \ldots ,t)$$

The ${𝓎}^{th}$ row ($\varphi_{𝓎1}$, $\varphi_{𝓎1}$, …, $\varphi_{𝓎t}$) of the membership degree matrix φ = ($\varphi_{𝓎𝓏}$) txt represents the degree that the rank of the ${𝓎}^{th}$ alternative belongs to k as shown in Equation (35).

(35) $$0\leq \varphi_{𝓎𝓏}\;\leq 1\;\mathrm{and}\;\sum_{𝓏=1}^{k}\varphi_{𝓎𝓏}=1.$$

Step 5.5: Calculate the final rank index $P_{𝓎}$ of the ${𝓎}^{th}$ alternative (𝓎=1,2,…,t), where $P_{𝓎}$ is calculated as per Equation (36).

(36) $$P_{𝓎}=\overset{t}{\underset{𝓏=1}{\sum }}𝓏\times \varphi_{𝓎𝓏}$$

Step 5.6: Determine final ranks (with minimum final rank index) r01, r02, …, r0t of the alternatives of TKR material in the ascending order based on the values of $P_{1},P_{2,}P_{3},\ldots \ldots P_{t}.$

## 3. Selection of Femoral Component (FC) Material for Total Knee Replacement (TKR)

**Figure 3:**
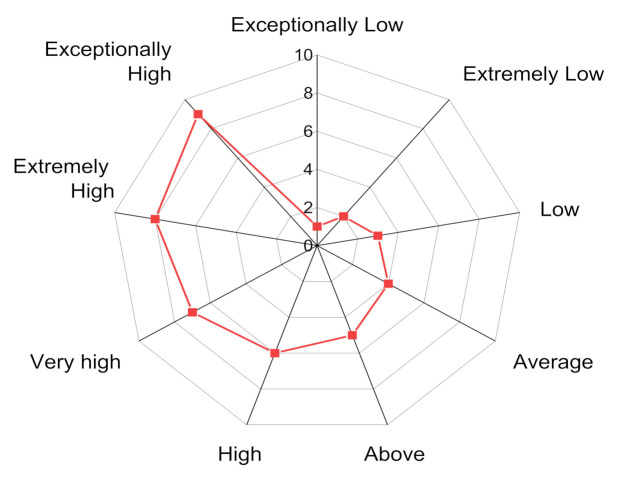
Qualitative degree of the attribute in the format of a 9-point scale.

The various attributes considered in the decision-making, such as cost, density and modulus of elasticity, are tabulated in Table 1 from 1_ps_ to 8_ps_. The eleven alternatives of FC material for TKR from 1_m_ to 11_m_ are shown in Figure 2. Figure 3 represents a qualitative degree of FC material of TKR attribute in a 9-point scale format. Table 2 is a decision matrix as per Equation (1). All the calculations were completed on Excel (MS Office) for up to four decimal places.

The equal weight method was used to attain the weights of importance, according to Step 3. The weights of each attribute are represented in Table 3.

The ranks of each alternative were computed using different methods, according to Step 4. Firstly, the SAW method was executed using Equation (11). The weighted sum for each alternative was calculated using each attribute’s optimal weight, as shown in Table 4. Similarly, the WPM method was executed using Equation (13), and the results are shown in Table 5.

The dual comparative significance of the alternative, i.e., performance index (Q_i_) based upon SAW and WPM techniques, was calculated, as shown in Equation (14). The final optimal assessment was executed using Equation (15), and the performance index of each alternative is shown in Table 6. 

According to all the criteria, the average solution (AV_ij_) of each attribute, 1_ps_ to 8_ps_, was calculated per Equation (16). Positive–negative-distance-based normalization was utilized in the EDAS. The positive distance from average ($PDA_{ij}$) was executed using Equation (17) for beneficial attributes and Equation (18) for nonbeneficial attributes and shown in Table 7. The negative distance from average $\left(PDA_{ij}\right)$ was executed using Equation (19) for beneficial attributes and Equation (20) for nonbeneficial attributes. The weighted sum of 𝒫𝒟𝒜 and 𝒩𝒟𝒜 is obtained from the average matrix from Equations (21) and (22). The normalized values of $SP_{i}$ are obtained from Equation (23) and $SN_{i}$ from Equation (24) for all alternatives. The appraisal score index AS_i_ for all alternatives was obtained by Equation (25) and is shown in Table 7. Finally, the optimal results from the TOPSIS method were obtained by calculating the ideal best (Z^+^) and ideal worst (Z^−^) solutions with the help of Equations (27) and (28), respectively. Here, Z^+^ and Z^−^ solutions are the utmost and least values amongst all response values. The final ranks, obtained using the different methods, are shown in Table 8.

The combined results of all the methods, including WSM, WPM, WASPAS, EDAS, and TOPSIS, are represented in Table 9.

The constitute rank frequency number of each alternative was calculated using Equation (33) and Step 5.3., and they are represented in Table 10. A membership degree is constituted using Equation (34) and Step 5.4. The final rank index of each alternative was obtained using Equation (36) and Step 5.5 from the DoM technique. The final ranks of each alternative were calculated accordingly and are represented in Figure 3. The ranks of FC material of TKR, assigned by different MCDM methods, and final ranks with DoM can be seen in Table 11. The first rank goes to 4_m_ Porous NiTi shape memory alloy, followed by 7_m_ Ti alloys (Ti–6Al–4V).

**Figure 4:**
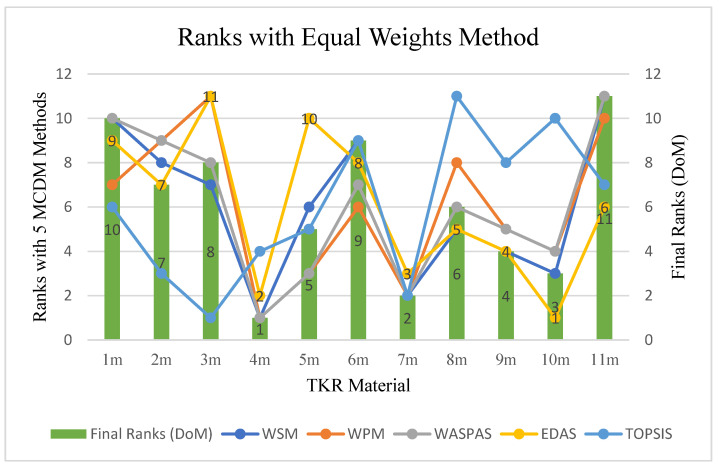
Ranks of FC material for TKR by different MCDM methods and DoM with EWM.

When considering EWM, femoral component material 4_m_ came out in first place, followed by 7_m_ and 10_m_, as shown in Table 11, as different MCDM methodologies have their own features. Thus, the variation of ranks with the variation in methods is presented in Figure 4. The coloured lines represent different methodologies, while the bars represent the final ranks calculated using the DoM technique.

## 4. Sensitivity Analysis

Sensitivity analysis is the review of the uncertainty in the output of a mathematical decision-making model or system to various risks and changes in its inputs. This analysis helps check the results’ consistency as to whether the model or system works in most conditions, favourable or unfavourable. The positive results obtained from the sensitivity analysis indicates the durability and robustness of the solution. The present study’s sensitivity analysis was executed by considering objective weights with the standard deviation method ‘SDM’, the entropy weights technique ‘EWT’, and the subjective weights with fuzzy analytical hierarchy process (FAHP) considered from the research [4]. The ranks by different MCDM methods, with SDM, EWT and Fuzzy AHP, are shown in Appendix A (Table A1, Table A3 and Table A5). The final rank index achieved with DoM, with SDM, EWT and Fuzzy AHP, is shown in Appendix A (Table A2, Table A4 and Table A6, respectively). The calculations were completed in a similar manner to those done for EWM, from Step 1 to Step 5.6. The ranks of FC material for TKR by different MCDM methods and DoM with SDM, EWT and fuzzy AHP are shown in 5–6, respectively.

**Figure 5:**
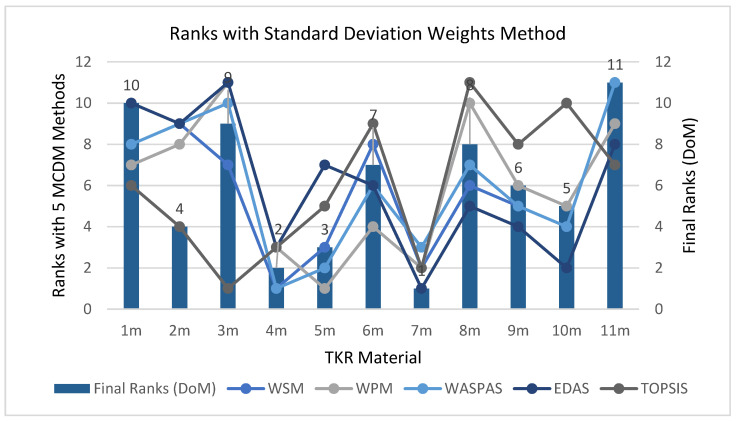
Ranks of FC material for TKR by different MCDM methods and DoM with SDM.

A similar analysis was done by changing weightage, as done before in Figure 4, but with SDM as the weightage. A high variation can be seen in 2_m_ and 5_m_, as in Figure 4, 2_m_ attains rank 7, but in Figure 5, it rises three ranks to rank 4; similarly, 5_m_ attains rank 5 in 4. but improves two ranks to rank 3 in Figure 5.

**Figure 6:**
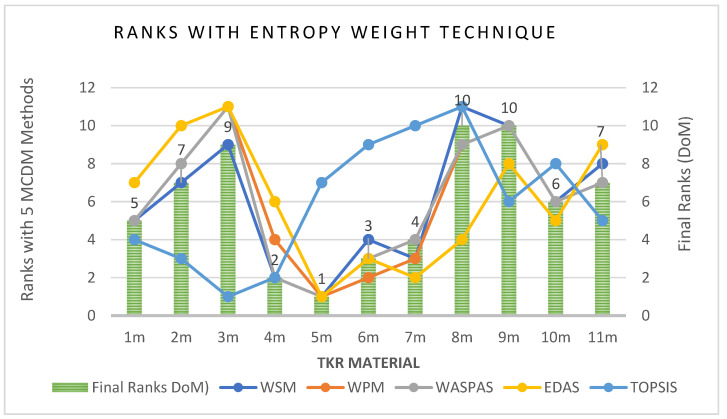
Ranks of FC material for TKR by different MCDM methods and DoM with EWT.

**Figure 7:**
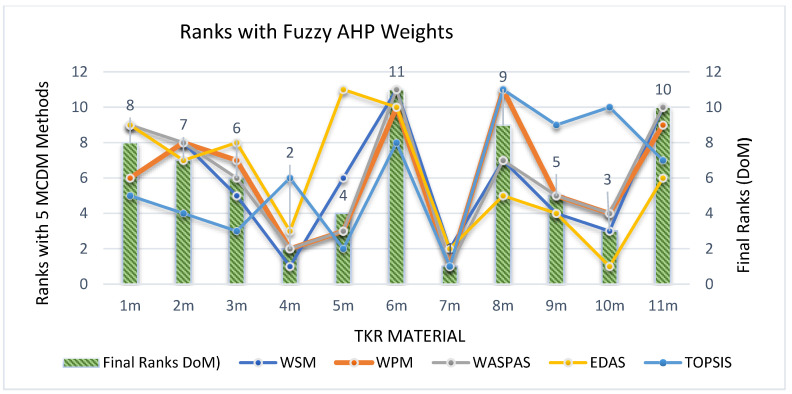
Ranks of FC material for TKR by different MCDM methods and DoM with Fuzzy AHP.

Correspondingly, with EWT and FAHP, the materials 6_m_ and 9_m_ show a sharp change as 6_m_ attains rank 3 in Figure 6**,** but falls to rank 11 in Figure 7. Similarly, 9_m_ in Figure 6 shows as rank 10, but it improves to rank 5 in Figure 7. Likewise, considering 1_m_, there is a drastic change in rank as it attains rank 5 with EWT, as shown in Figure 6 but slips to rank 8 when taking FAHP as weightage in Figure 7.

**Figure 8:**
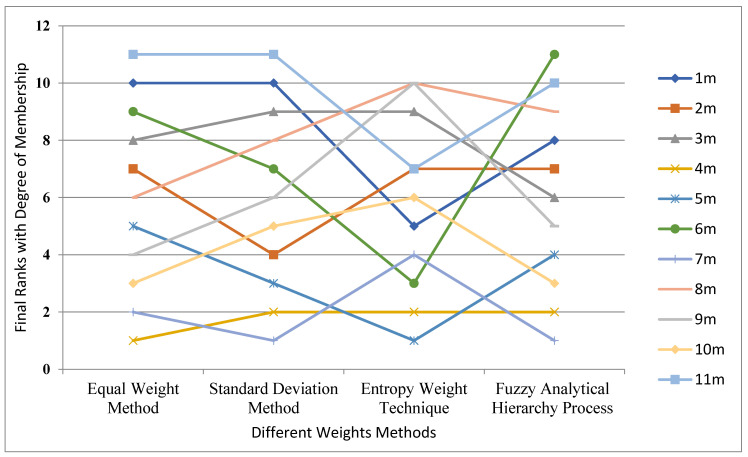
Final ranks with DoM with all weighting criteria.

The final ranks with DoM, with all weighting criteria viz. EWM, SDM, EWT and fuzzy AHP, are shown in Figure 8. The FC material for TKR 4_m_ Porous NiTi shape memory alloy is ranked first with EWM and at the second rank with SDM, EWT and fuzzy AHP weight methods. The 4_m_ Porous NiTi shape memory alloy was also assigned rank 1 by [4,45]. For FC material for TKR, 7_m_ Ti alloys (Ti–6Al–4V) are at rank 1 with SDM and fuzzy AHP weight methods, rank 2 by EWM, and rank 4 by EWT. High consistency can be seen in 8. Although 4_m_ shifts to the second rank by changing the weightage, it remains at the second position, proving the top-ranked material performs extraordinarily in different circumstances.

## 5. Conclusions

The appropriate femoral component (FC) material selection for total knee replacement (TKR) is the tactical aim of researchers and other decision-makers. A hybrid multicriteria decision-making approach was developed and applied to select FC material for TKR. The best alternative decision was made based upon five different MCDM techniques with EWM; these techniques assign ranks with diverse principles. The final rank was achieved with a degree of membership while combining the rank results of the five MCDM methods. The 11 available FC materials for TKR were considered with 8 significant attributes. The sensitivity analysis was conducted with objective and subjective weights. The sensitivity analysis indicated that the FC material using TKR Ti alloys (Ti–6Al–4V) is at rank 1 with the SDM and fuzzy AHP weight methods, rank 2 with EWM and at rank 4 with EWT. It has a USD 105 (Mg/m^3^) price, with properties such as density 4.5 g/cc, modulus of elasticity 100 GPa, tensile strength 550 MPa, elongation 54%, exceptionally high corrosion resistance, above-average wear resistance, and high osseointegration. At the same time, the Zr alloy (Zr-2.5Nb) came out as last rank with EWM and SDM and second-to-last rank when taking FAHP as weightage. Overall, the proposed methodology provides robust results with the DoM; it has more statistical simplicity and the potential to produce more accurate results.

Furthermore, the range of biomaterials can be expanded with more alternatives and attributes, considering not only the femoral component but also components for hip joints, scaffolds, orthopaedic accessories, and stents. More studies are being carried out on using titanium-based alloys for knee replacements, and researchers have developed new high-strength low-elastic β-phase titanium alloys. This multi-criteria decision-making approach can be useful for comparing recently developed materials with existing ones. Other parts of knee prostheses, such as tibial trays, can be a research subject. However, some new attributes, such as porosity and manufacturability, must be considered depending upon the application as the tibial component varies from the femoral component from mechanical and biological points of view.

## Figures and Tables

**Table 1:** Significant attributes for FC material for TKR.

Sr. No.	Attribute	Abbreviation of Attribute
1	Cost (Mg/m^3^)	1_ps_
2	Density (g/cc)	2_ps_
3	Elastic Modulus (GPa)	3_ps_
4	Tensile Strength (MPa)	4_ps_
5	Elongation (%)	5_ps_
6	Corrosion resistance	6_ps_
7	Wear resistance	7_ps_
8	Osseointegration	8_ps_

**Table 2:** Decision matrix of applicant TKR material.

Material	1_ps_	2_ps_	3_ps_	4_ps_	5_ps_	6_ps_	7_ps_	8_ps_
1_m_	74	8.3	240	655	20	7	8	6
2_m_	103	9.13	240	896	20	7	8	6
3_m_	450	6.45	75	960	15.5	8	9	4
4_m_	370	4.3	15	1000	12	7	9	9
5_m_	15	8	200	517	40	6	5	5
6_m_	31	8	200	862	12	6	7	5
7_m_	105	4.5	100	550	54	9	5	7
8_m_	145	4.45	112	900	6	9	6	7
9_m_	191	4.43	112	985	12	9	6	7
10_m_	165	4.52	110	1000	12	9	6	7
11_m_	216	6.44	98	510	20	7	7	7

**Table 3:** Weightage by EWM.

Weight	1_ps_	2_ps_	3_ps_	4_ps_	5_ps_	6_ps_	7_ps_	8_ps_
EWM %	12.5	12.5	12.5	12.5	12.5	12.5	12.5	12.5

**Table 4:** Weighted sum method with EWM.

Material	1_ps_	2_ps_	3_ps_	4_ps_	5_ps_	6_ps_	7_ps_	8_ps_	SUM	Rank
1_m_	0.0253	0.0648	0.0078	0.0819	0.0463	0.0972	0.1111	0.0833	0.5177	10
2_m_	0.0182	0.0589	0.0078	0.1120	0.0463	0.0972	0.1111	0.0833	0.5349	8
3_m_	0.0042	0.0833	0.0250	0.1200	0.0359	0.1111	0.1250	0.0556	0.5600	7
4_m_	0.0051	0.1250	0.1250	0.1250	0.0278	0.0972	0.1250	0.1250	0.7551	1
5_m_	0.1250	0.0672	0.0094	0.0646	0.0926	0.0833	0.0694	0.0694	0.5810	6
6_m_	0.0605	0.0672	0.0094	0.1078	0.0278	0.0833	0.0972	0.0694	0.5226	9
7_m_	0.0179	0.1194	0.0188	0.0688	0.1250	0.1250	0.0694	0.0972	0.6415	2
8_m_	0.0129	0.1208	0.0167	0.1125	0.0139	0.1250	0.0833	0.0972	0.5824	5
9_m_	0.0098	0.1213	0.0167	0.1231	0.0278	0.1250	0.0833	0.0972	0.6043	4
10_m_	0.0114	0.1189	0.0170	0.1250	0.0278	0.1250	0.0833	0.0972	0.6057	3
11_m_	0.0087	0.0835	0.0191	0.0638	0.0463	0.0972	0.0972	0.0972	0.5130	11

**Table 5:** Weighted product method with EWM.

Material	1_ps_	2_ps_	3_ps_	4_ps_	5_ps_	6_ps_	7_ps_	8_ps_	Product	Rank
1_m_	0.8191	0.9211	0.7071	0.9485	0.8832	0.9691	0.9854	0.9506	0.4057	7
2_m_	0.7860	0.9102	0.7071	0.9864	0.8832	0.9691	0.9854	0.9506	0.4000	9
3_m_	0.6537	0.9506	0.8178	0.9949	0.8555	0.9854	1.0000	0.9036	0.3851	11
4_m_	0.6699	1.0000	1.0000	1.0000	0.8286	0.9691	1.0000	1.0000	0.5379	1
5_m_	1.0000	0.9253	0.7234	0.9208	0.9632	0.9506	0.9292	0.9292	0.4872	3
6_m_	0.9133	0.9253	0.7234	0.9816	0.8286	0.9506	0.9691	0.9292	0.4256	6
7_m_	0.7841	0.9943	0.7889	0.9280	1.0000	1.0000	0.9292	0.9691	0.5139	2
8_m_	0.7531	0.9957	0.7778	0.9869	0.7598	1.0000	0.9506	0.9691	0.4029	8
9_m_	0.7276	0.9963	0.7778	0.9981	0.8286	1.0000	0.9506	0.9691	0.4295	5
10_m_	0.7410	0.9938	0.7795	1.0000	0.8286	1.0000	0.9506	0.9691	0.4382	4
11_m_	0.7165	0.9508	0.7909	0.9193	0.8832	0.9691	0.9691	0.9691	0.3981	10

**Table 6:** WASPAS with EWM.

Material	WSM	WPM	Var Qi WSM	Var Qi WPM	SUM	Optimal ƛ	Qi WASPAS	Rank
1_m_	0.5177	0.4057	0.0049	0.0025	0.0074	0.3389	0.4437	10
2_m_	0.5349	0.4000	0.0047	0.0025	0.0072	0.3496	0.4472	9
3_m_	0.5600	0.3851	0.0046	0.0026	0.0072	0.3673	0.4494	8
4_m_	0.7551	0.5379	0.0070	0.0032	0.0102	0.3168	0.6067	1
5_m_	0.5810	0.4872	0.0045	0.0026	0.0071	0.3660	0.5216	3
6_m_	0.5226	0.4256	0.0051	0.0024	0.0072	0.3344	0.4580	7
7_m_	0.6415	0.5139	0.0048	0.0029	0.0077	0.3755	0.5618	2
8_m_	0.5824	0.4029	0.0045	0.0025	0.0070	0.3578	0.4671	6
9_m_	0.6043	0.4295	0.0046	0.0024	0.0070	0.3468	0.4902	5
10_m_	0.6057	0.4382	0.0046	0.0024	0.0070	0.3459	0.4961	4
11_m_	0.5130	0.3981	0.0049	0.0025	0.0075	0.3406	0.4372	11

**Table 7:** EDAS with EWM.

Material	SP_i_	SN_i_	NSP_i_	NSN_i_	AS_i_	Rank
1_m_	0.0902	0.1788	0.2997	0.3774	0.3386	9
2_m_	0.0977	0.1724	0.3247	0.3997	0.3622	7
3_m_	0.1489	0.2873	0.4950	0.0000	0.2475	11
4_m_	0.3009	0.2094	1.0000	0.2711	0.6356	2
5_m_	0.1139	0.2263	0.3787	0.2123	0.2955	10
6_m_	0.1221	0.1984	0.4058	0.3095	0.3576	8
7_m_	0.1506	0.0739	0.5004	0.7426	0.6215	3
8_m_	0.1412	0.1045	0.4694	0.6361	0.5528	5
9_m_	0.1500	0.0834	0.4985	0.7095	0.6040	4
10_m_	0.1580	0.0676	0.5253	0.7646	0.6449	1
11_m_	0.0494	0.0965	0.1643	0.6641	0.4142	6

**Table 8:** TOPSIS with EWM.

Material	Si +	Si −	sum	Pi	Rank
1_m_	0.0887	0.0681	0.1569	0.4344	6
2_m_	0.0835	0.0727	0.1562	0.4656	3
3_m_	0.0793	0.0865	0.1658	0.5218	1
4_m_	0.0914	0.0765	0.1679	0.4558	4
5_m_	0.0907	0.0729	0.1636	0.4457	5
6_m_	0.1036	0.0551	0.1587	0.3471	9
7_m_	0.0816	0.0818	0.1634	0.5008	2
8_m_	0.1027	0.0443	0.1470	0.3014	11
9_m_	0.0918	0.0514	0.1432	0.3589	8
10_m_	0.0942	0.0488	0.1430	0.3414	10
11_m_	0.0823	0.0524	0.1347	0.3889	7

**Table 9:** Ranks by different MCDM methods with EWM.

Method	WSM	WPM	WASPAS	EDAS	TOPSIS
1_m_	10	7	10	9	6
2_m_	8	9	9	7	3
3_m_	7	11	8	11	1
4_m_	1	1	1	2	4
5_m_	6	3	3	10	5
6_m_	9	6	7	8	9
7_m_	2	2	2	3	2
8_m_	5	8	6	5	11
9_m_	4	5	5	4	8
10_m_	3	4	4	1	10
11_m_	11	10	11	6	7

**Table 10:** Rank frequency number with EWM.

Material	1	2	3	4	5	6	7	8	9	10	11
1_m_	0	0	0	0	0	1	1	0	1	2	0
2_m_	0	0	1	0	0	0	1	1	2	0	0
3_m_	1	0	0	0	0	0	1	1	0	0	2
4_m_	3	1	0	1	0	0	0	0	0	0	0
5_m_	0	0	2	0	1	1	0	0	0	1	0
6_m_	0	0	0	0	0	1	1	1	2	0	0
7_m_	0	4	1	0	0	0	0	0	0	0	0
8_m_	0	0	0	0	2	1	0	1	0	0	1
9_m_	0	0	0	2	2	0	0	1	0	0	0
10_m_	1	0	1	2	0	0	0	0	0	1	0
11_m_	0	0	0	0	0	1	1	0	0	1	2

**Table 11:** Final rank index with EWM.

Material	1	2	3	4	5	6	7	8	9	10	11	SUM	Rank
1_m_	0.00	0.00	0.00	0.00	0.00	1.20	1.40	0.00	1.80	4.00	0.00	8.40	10
2_m_	0.00	0.00	0.60	0.00	0.00	0.00	1.40	1.60	3.60	0.00	0.00	7.20	7
3_m_	0.20	0.00	0.00	0.00	0.00	0.00	1.40	1.60	0.00	0.00	4.40	7.60	8
4_m_	0.60	0.40	0.00	0.80	0.00	0.00	0.00	0.00	0.00	0.00	0.00	1.80	1
5_m_	0.00	0.00	1.20	0.00	1.00	1.20	0.00	0.00	0.00	2.00	0.00	5.40	5
6_m_	0.00	0.00	0.00	0.00	0.00	1.20	1.40	1.60	3.60	0.00	0.00	7.80	9
7_m_	0.00	1.60	0.60	0.00	0.00	0.00	0.00	0.00	0.00	0.00	0.00	2.20	2
8_m_	0.00	0.00	0.00	0.00	2.00	1.20	0.00	1.60	0.00	0.00	2.20	7.00	6
9_m_	0.00	0.00	0.00	1.60	2.00	0.00	0.00	1.60	0.00	0.00	0.00	5.20	4
10_m_	0.20	0.00	0.60	1.60	0.00	0.00	0.00	0.00	0.00	2.00	0.00	4.40	3
11_m_	0.00	0.00	0.00	0.00	0.00	1.20	1.40	0.00	0.00	2.00	4.40	9.00	11

## Data Availability

The data presented in this study are available on request from the corresponding author.

## Appendix A

**Table A1:** Ranks by different MCDM methods with SDM.

METHOD	WSM	WPM	WASPAS	EDAS	TOPSIS
1_m_	10	7	8	10	6
2_m_	9	8	9	9	4
3_m_	7	11	10	11	1
4_m_	1	3	1	3	3
5_m_	3	1	2	7	5
6_m_	8	4	6	6	9
7_m_	2	2	3	1	2
8_m_	6	10	7	5	11
9_m_	5	6	5	4	8
10_m_	4	5	4	2	10
11_m_	11	9	11	8	7

**Table A2:** Final rank index with SDM.

Material	1	2	3	4	5	6	7	8	9	10	11	SUM	Rank
1_m_	0.00	0.00	0.00	0.00	0.00	1.20	1.40	1.60	0.00	4.00	0.00	8.20	10
2_m_	0.00	0.00	1.80	0.80	0.00	0.00	0.00	1.60	0.00	0.00	0.00	4.20	4
3_m_	0.20	0.00	0.00	0.00	0.00	0.00	1.40	0.00	0.00	2.00	4.40	8.00	9
4_m_	0.40	0.00	1.80	0.00	0.00	0.00	0.00	0.00	0.00	0.00	0.00	2.20	2
5_m_	0.20	0.40	0.60	0.00	1.00	0.00	1.40	0.00	0.00	0.00	0.00	3.60	3
6_m_	0.00	0.00	0.00	0.80	0.00	2.40	0.00	1.60	1.80	0.00	0.00	6.60	7
7_m_	0.20	1.20	0.60	0.00	0.00	0.00	0.00	0.00	0.00	0.00	0.00	2.00	1
8_m_	0.00	0.00	0.00	0.00	1.00	1.20	1.40	0.00	0.00	2.00	2.20	7.80	8
9_m_	0.00	0.00	0.00	0.80	2.00	1.20	0.00	1.60	0.00	0.00	0.00	5.60	6
10_m_	0.00	0.40	0.00	1.60	1.00	0.00	0.00	0.00	0.00	2.00	0.00	5.00	5
11_m_	0.00	0.00	0.00	0.00	0.00	0.00	1.40	1.60	1.80	0.00	4.40	9.20	11

**Table A3:** Ranks by different MCDM methods with EWT.

METHOD	WSM	WPM	WASPAS	EDAS	TOPSIS
1_m_	5	5	5	7	4
2_m_	7	8	8	10	3
3_m_	9	11	11	11	1
4_m_	2	4	2	6	2
5_m_	1	1	1	1	7
6_m_	4	2	3	3	9
7_m_	3	3	4	2	10
8_m_	11	9	9	4	11
9_m_	10	10	10	8	6
10_m_	6	6	6	5	8
11_m_	8	7	7	9	5

**Table A4:** Final rank index with EWT.

Material	1	2	3	4	5	6	7	8	9	10	11	SUM	Rank
1_m_	0.00	0.00	0.00	0.80	3.00	0.00	1.40	0.00	0.00	0.00	0.00	5.20	5
2_m_	0.00	0.00	0.60	0.00	0.00	0.00	1.40	3.20	0.00	2.00	0.00	7.20	7
3_m_	0.20	0.00	0.00	0.00	0.00	0.00	0.00	0.00	1.80	0.00	6.60	8.60	9
4_m_	0.00	1.20	0.00	0.80	0.00	1.20	0.00	0.00	0.00	0.00	0.00	3.20	2
5_m_	0.80	0.00	0.00	0.00	0.00	0.00	1.40	0.00	0.00	0.00	0.00	2.20	1
6_m_	0.00	0.40	1.20	0.80	0.00	0.00	0.00	0.00	1.80	0.00	0.00	4.20	3
7_m_	0.00	0.40	1.20	0.80	0.00	0.00	0.00	0.00	0.00	2.00	0.00	4.40	4
8_m_	0.00	0.00	0.00	0.80	0.00	0.00	0.00	0.00	3.60	0.00	4.40	8.80	10
9_m_	0.00	0.00	0.00	0.00	0.00	1.20	0.00	1.60	0.00	6.00	0.00	8.80	10
10_m_	0.00	0.00	0.00	0.00	1.00	3.60	0.00	1.60	0.00	0.00	0.00	6.20	6
11m	0.00	0.00	0.00	0.00	1.00	0.00	2.80	1.60	1.80	0.00	0.00	7.20	7

**Table A5:** Ranks by different MCDM methods with fuzzy AHP.

Method	WSM	WPM	WASPAS	EDAS	TOPSIS
1_m_	9	6	9	9	5
2_m_	8	8	8	7	4
3_m_	5	7	6	8	3
4_m_	1	2	2	3	6
5_m_	6	3	3	11	2
6_m_	11	10	11	10	8
7_m_	2	1	1	2	1
8_m_	7	11	7	5	11
9_m_	4	5	5	4	9
10_m_	3	4	4	1	10
11_m_	10	9	10	6	7

**Table A6:** Final rank index with fuzzy AHP.

Material	1	2	3	4	5	6	7	8	9	10	11	SUM	Rank
1_m_	0.00	0.00	0.00	0.00	1.00	1.20	0.00	0.00	5.40	0.00	0.00	7.60	8
2_m_	0.00	0.00	0.00	0.80	0.00	0.00	1.40	4.80	0.00	0.00	0.00	7.00	7
3_m_	0.00	0.00	0.60	0.00	1.00	1.20	1.40	1.60	0.00	0.00	0.00	5.80	6
4_m_	0.20	0.80	0.60	0.00	0.00	1.20	0.00	0.00	0.00	0.00	0.00	2.80	2
5_m_	0.00	0.40	1.20	0.00	0.00	1.20	0.00	0.00	0.00	0.00	2.20	5.00	4
6_m_	0.00	0.00	0.00	0.00	0.00	0.00	0.00	1.60	0.00	4.00	4.40	10.00	11
7_m_	0.60	0.80	0.00	0.00	0.00	0.00	0.00	0.00	0.00	0.00	0.00	1.40	1
8_m_	0.00	0.00	0.00	0.00	1.00	0.00	2.80	0.00	0.00	0.00	4.40	8.20	9
9_m_	0.00	0.00	0.00	1.60	2.00	0.00	0.00	0.00	1.80	0.00	0.00	5.40	5
10_m_	0.20	0.00	0.60	1.60	0.00	0.00	0.00	0.00	0.00	2.00	0.00	4.40	3
11_m_	0.00	0.00	0.00	0.00	0.00	1.20	1.40	0.00	1.80	4.00	0.00	8.40	10

## References

[1] Bahraminasab M., Jahan A. (2011). Material selection for femoral component of total knee replacement using comprehensive VIKOR. Mater. Des..

[2] Bahraminasab M., Farahmand F. (2017). State of the art review on design and manufacture of hybrid biomedical materials: Hip and knee prostheses. Part H J. Eng. Med..

[3] Bahraminasab M., Hassan M.R., Sahari B. (2010). Metallic biomaterials of Knee and hip—A review. Trends Biomater. Artif. Organs.

[4] Kabir G., Lizu A. (2016). Material selection for femoral component of total knee replacement integrating fuzzy AHP with PROMETHEE. J. Intell. Fuzzy Syst..

[5] Van den Heever D., Scheffer C., Erasmus P., Dillon E. (2011). Method for selection of femoral component in total knee arthroplasty (tka). Australas Phys. Eng. Sci. Med..

[6] Buechel F.F., Pappas M.J. (2015). Principles of Human Joint Replacement: Design and Clinical Application.

[7] Bal S. (2013). Your Guide to Knee Replacement Surgery.

[8] Elsayed B.M.E. (2016). The Effect of Changing the Shape and Material of Tibial Component on the Performance of Total Knee Replacement. Master’s Thesis.

[9] Zavatsky A., O’Connor J.J. (1992). A model of human knee ligaments in the sagittal plane: Part 2: Fibre recruitment under load. Part H J. Eng. Med..

[10] Zavatsky A., O’Connor J.J. (1992). A Model of human knee ligaments in the sagittal plane: Part 1: Response to passive flexion. Part H J. Eng. Med..

[11] Scanlon V.C., Sanders T. (2018). Essentials of Anatomy and Physiology.

[12] Tortora G.J., Derrickson B.H. (2018). Principles of Anatomy and Physiology.

[13] Kandadai R., Harsha S. (2016). Study of Stress Levels in Various Materials in Total Knee Replacement under Static Condition. IOSR J. Mech. Civ. Eng..

[14] Johnson G.R., Revell P.A. (2008). 1—Biomechanics of joints. Joint Replacement Technology.

[15] Willing R., Kim I.Y. (2008). Three dimensional shape optimization of total knee replacements for reduced wear. Struct. Multidiscip. Optim..

[16] Sivarasu S., Mathew L. (2009). Structural Responses of a Novel High Flexion Knee (Ss316–Uhmwpe) Used in Total Knee Arthroplasty Using Finite Element Analysis. Biophys. Rev. Lett..

[17] Geetha M., Singh A.K., Asokamani R., Gogia A.K. (2009). Ti based biomaterials, the ultimate choice for orthopaedic implants—A review. Prog. Mater. Sci..

[18] Farag M.M. (2013). Materials and Process Selection for Engineering Design.

[19] Long M., Rack H. (1998). Titanium alloys in total joint replacement—A materials science perspective. Biomaterials.

[20] Wang K. (1996). The use of titanium for medical applications in the USA. Mater. Sci. Eng. A.

[21] Okazaki Y., Gotoh E. (2005). Comparison of metal release from various metallic biomaterials in vitro. Biomaterials.

[22] Ramsden J., Allen D., Stephenson D., Alcock J., Peggs G., Fuller G., Goch G. (2007). The Design and Manufacture of Biomedical Surfaces. CIRP Ann..

[23] Neailey K., Pond R. (1982). Metal implants. Mater. Des..

[24] Williams D.F. (2008). On the mechanisms of biocompatibility. Biomaterials.

[25] Viceconti M., Muccini R., Bernakiewicz M., Baleani M., Cristofolini L. (2000). Large-sliding contact elements accurately predict levels of bone–implant micromotion relevant to osseointegration. J. Biomech..

[26] Swami V., Vijayaraghavan V., Swami V. (2016). Current trends to measure implant stability. J. Indian Prosthodont. Soc..

[27] Sennerby L., Roos J. (1999). Surgical determinants of clinical success of osseointegrated oral implants: A review of the literature. Int. J. Prosthodont..

[28] Helsen J.A., Jürgen Breme H. (1998). Metals as Biomaterials.

[29] Brunski J.J.B.S. (1996). An Introduction to Material in Medicine.

[30] Chen Q., Thouas G.A. (2015). Metallic implant biomaterials. Mater. Sci. Eng. R Rep..

[31] Kumar S., Dewan L., Dewan L., Bansal R.C., Kumar Kalla U. (2021). Selection of the Best Material for Coating on Solar Cell and Optical Filter. Advances in Renewable Energy and Sustainable Environment, Proceedings of the National Conference on Renewable Energy and Sustainable Environment, NCRESE 2019, Kurukshetra, India, 30–31 August 2019.

[32] Kumar R., Singal S.K. (2015). Penstock material selection in small hydropower plants using MADM methods. Renew. Sustain. Energy Rev..

[33] Rahman M.A., Pereda V.A. (2016). Freight transport and logistics evaluation using entropy technique integrated to topsis algorithm. Design Solutions for User-Centric Information Systems.

[34] Mathew M., Sahu S. (2018). Comparison of new multi-criteria decision making methods for material handling equipment selection. Manag. Sci. Lett..

[35] Yazdani M. (2018). New approach to select materials using MADM tools. IJSBR.

[36] Ghorabaee M.K., Zavadskas E.K., Olfat L., Turskis Z. (2015). Multi-Criteria Inventory Classification Using a New Method of Evaluation Based on Distance from Average Solution (EDAS). Informatica.

[37] Ghorabaee M.K., Amiri M., Zavadskas E.K., Turskis Z. (2017). Multi-criteria group decision-making using an extended edas method with interval type-2 fuzzy sets. Ekon. Manag..

[38] Dhanalakshmi C.S., Madhu P., Karthick A., Mathew M., Kumar R.V. (2020). A comprehensive MCDM-based approach using TOPSIS and EDAS as an auxiliary tool for pyrolysis material selection and its application. Biomass Convers. Biorefinery.

[39] Kumar R., Singh S. (2020). Selection of vacuum cleaner with Technique for Order Preference by Similarity to Ideal Solution method based upon multi-criteriadecision-making theory. Meas. Control..

[40] Kumar R., Bhattacherjee A., Singh A.D., Singh S., Pruncu I.C. (2020). Selection of portable hard disk drive based upon weighted aggregated sum product assessment method: A case of Indian market. Meas. Control..

[41] Rohit Dubey R.K., Bilga P.S., Singh S. (2020). Selection of Inverter Technology Air Conditioner: An Evaluation Based on Distance from Average Solution. Sustainable Development through Engineering Innovations, Proceedings of the International Conference on Sustainable Development through Engineering Innovations, Punjab, India, 17–19 September 2020.

[42] Raman Kumar H.K. (2020). Harpreet Singh, Selection of Mobile Phone with Multi Criteria Decision Making Approach: A Case Study. Future of Business through Innovations.

[43] Kumar R., Kumar R., Soni G., Chhabra S. (2013). Optimization of process parameters during CNC turning by using AHP & VIKOR method. Int. J. Eng. Res. Technol..

[44] Kumar R., Kumar R., Rai J.S., Virk N.S. (2013). Analysis the effects of process parameters in EN24 alloy steel during CNC turning by using MADM. Int. J. Innov. Res. Sci. Eng. Technol..

[45] Yang W.-C., Chon S.-H., Choe C.-M., Kim U.-H. (2019). Materials Selection Method Combined with Different MADM Methods. J. Artif. Intell..

[46] Kumar R., Bilga P.S., Singh S. (2017). Multi objective optimization using different methods of assigning weights to energy consumption responses, surface roughness and material removal rate during rough turning operation. J. Clean. Prod..

[47] Singh G., Singh G., Prakash C., Kumar R., Kumar R., Ramakrishna S. (2020). Characterization of three-dimensional printed thermal-stimulus polylactic acid-hydroxyapatite-based shape memory scaffolds. Polym. Compos..

[48] Singh H., Kumar R. Selection of material for bicycle chain in indian scenario using madm approach. Proceedings of the 2012 World Congress on Engineering, WCE 2012.

[49] Kumar R., Singh S., Bilga P.S., Jatin, Singh J., Singh S., Scutaru M.-L., Pruncu C.I. (2021). Revealing the benefits of entropy weights method for multi-objective optimization in machining operations: A critical review. J. Mater. Res. Technol..

[50] Zavadskas E.K., Turskis Z., Antucheviciene J., Zakarevicius A. (2012). Optimization of weighted aggregated sum product assessment. Elektron. Elektrotechnika.

